# Colorectal Cancer Screening Communication and Completion Among Black and Hispanic Primary Care Patients

**DOI:** 10.21203/rs.3.rs-10831545/v1

**Published:** 2026-08-28

**Authors:** Jessica N Rivera Rivera, Katarina E AuBuchon, Laura C Schubel, Jennifer Tran, Allan Fong, Katharine Adams, Mihriye Mete, Melanie Grady, Jessica E. Galarraga, Hannah Arem

**Affiliations:** MedStar Health Research Institute; MedStar Health Research Institute; MedStar Health Research Institute; MedStar Washington Hospital Center; MedStar Health Research Institute; MedStar Health Research Institute; MedStar Health Research Institute; MedStar Health Institute for Quality and Safety; MedStar Health; MedStar Health Research Institute

**Keywords:** Colorectal cancer screening completion, patient-centered communication, Black patients, Hispanic patients, primary care

## Abstract

**Background.:**

Colorectal cancer (CRC) screening is recommended for all adults aged 45–75 years. However, CRC screening and outcome disparities exist by race and ethnicity.

**Objective.:**

To examine factors related to Black and Hispanic patients’ experiences regarding primary care provider CRC screening communication and completion.

**Design.:**

A cross-sectional study.

**Participants.:**

Black and Hispanic patients with a recent visit at seven primary care clinics in Washington, DC, and Maryland were identified from the electronic medical record.

**Main Measures.:**

Participants completed a 10-minute survey on CRC screening. We also collected demographics (age, sex, education, insurance), health literacy, and responses to the Patient-Centered Communication (PCC) scale about CRC screening (PCC; range 1 to 4, 4 = Always) and Distrust in the Healthcare System (range 1 to 4, 4 = greater distrust).

**Key Results.:**

Of the 913 patients we contacted, 278 (30%) agreed to participate, and 204 (70%) completed the survey. Mean age was 57 (SD=8.4), 77% were non-Hispanic Black, 62% female, 57% had a high school diploma or less, 46% had public insurance, and 20% completed the survey in Spanish. Patients reported high health literacy (*Ms*=4.03; SDs=1.36 & 1.47), high PCC (*M*=3.59; SD=0.59), and low distrust (*M*=2.03; SD=0.45). Patients reported that CRC screening was never discussed (13.7%), recommended but not completed (36.3%), previously screened but not up to date (15.2%), and up to date (34.8%). In the multivariate model, older age and health literacy were associated with CRC screening completion, and Hispanic ethnicity was associated with lower CRC screening.

**Conclusions.:**

Differences in CRC screening completion were documented by age, ethnicity, and health literacy. Tailoring outreach to younger, Hispanic, and lower health literate populations may improve CRC screening uptake and guideline-adherence.

## Background

In the U.S., colorectal cancer (CRC) is the second most common cause of cancer death.^1^ Significant unfair and avoidable differences in outcomes by racial group in CRC persist.^2^ Though the inequity is declining, Black people in the U.S. have higher CRC mortality.^3^ Although CRC screening is a key factor to reduce CRC mortality, Hispanic people in the U.S. demonstrate lower CRC screening than both Black and White people.^4^ Importantly, research demonstrates that these inequities are driven by social barriers, as controlling for factors such as income often mitigates CRC inequities, and interventions to provide transportation or cost coverage increase screening rates.^2^

Other modifiable and related factors that influence CRC screening include clinician-patient communication, health literacy, and medical trust or distrust. Research demonstrates that clinician-patient communication can influence future cancer screening plans and behavior,^5–7^ but there is limited research on clinician-patient communication specifically for Black and Hispanic patients. Understanding screening communication among Black and Hispanic patients is critical, because research indicates that cognitive biases can influence clinical communication.^8, 9^ For example, studies demonstrate that clinicians with high implicit bias scores receive higher communication ratings when speaking with White (vs. Black) standardized patients,^9^ and have shorter interactions and worse patient-centered communication scores with Black (vs. White) patients.^10^ Furthermore, Black patients experience worse clinical communication relative to their White counterparts.^11–13^

Research on clinician-patient communication with Hispanic patients is limited. Language is a well-known communication barrier for Hispanic patients who prefer Spanish^14^ and can result in lower quality clinical communication.^14, 15^ Another important component of communication is a patients’ health literacy, which can influence a patients’ confidence in communicating with their provider.^16^ Research on the relationship between health literacy and CRC screening is mixed, with some work demonstrating that high health literacy is related to higher screening completion, and other studies showing no relationship.^17^ Understanding this relationship with communication about screening can help identify patients who may require more education about CRC screening. More research is needed to understand how communication patterns may influence CRC screening for Black and Hispanic patients.

Medical trust is another driver of CRC disparities among Black and Hispanic patients.^18, 19^ Black and Hispanic patients’ distrust of healthcare is driven by experiences of discrimination; reflecting longstanding racialized barriers to care for both Black and Hispanic adults in the US.^20, 21^ Medical mistrust is a documented barrier to CRC screening among Black adults in the US,^18, 22^ and also predicts lower CRC screening among Hispanic adults.^23^ Thus, understanding how mistrust relates to CRC screening communication is a key factor to consider.

Understanding how these modifiable factors relate to CRC screening among Black and Hispanic patients offers a key potential area to target for future interventions to promote screening equity. We thus sought to identify sociodemographic (health literacy, racial group), cognitive (distrust), and communication factors that relate to CRC screening communication and completion among Black and Hispanic patients after a recent primary care visit.

## Methods

This survey was part of a larger stepped wedge, cluster randomized trial of 15 primary care clinics within a mid-Atlantic healthcare system (registered in clinicaltrials.gov NCT06401174 on May 6, 2024).^24^ The 15 clinics were selected to provide diversity in geography, baseline CRC screening rates, percent of patients with Medicaid, and disparities in CRC screening rates by race or ethnicity within clinics relative to White patients. We invited nine clinics that had not yet been randomized to the intervention arm to participate in the survey. One declined due to staffing turnover and very low volume at the time of the survey, and the other did not respond to outreach, leaving seven clinics available for the survey.

### Participants.

Patients aged 45 to 75 who were identified per the medical record as Non-Hispanic Black or African American (referred to as Black), or Hispanic or Latine (referred to as Hispanic), with an appointment completion at one of the seven primary care clinics within the prior two weeks were invited to participate. Research staff called patients to offer survey completion by phone or online via REDCap. The survey took approximately 10 minutes to complete. A total of 913 patients were contacted, 278 (30%) agreed to participate, and 204 (70%) of those patients completed the survey. After initial phone outreach, in an attempt to increase participation, 19 patients also received a letter with the survey QR code, and 2 completed the survey; letter outreach was abandoned due to low uptake. Of the 204 participants, 93% completed the survey via phone, and 7% completed the survey online from an emailed link. Participants received a $10 gift card or check for completing the survey.

### Measures.

Demographic data was obtained via self-report; race and ethnicity were reported from the medical record. Patients reported their demographics (sex, highest education level, insurance), and preferred language to complete the survey (English or Spanish). To assess health literacy, we used 2 of the 3 Health Literacy Screening questions: “How confident are you in filling out medical forms by yourself?” (Not at all = 1 to Extremely = 5; referred in this study as “Confident filling medical forms”) and “How often do you have someone help you read hospital materials?” (Always = 1 to Never = 5; referred in this study as “Does not need help reading hospital materials”).^25, 26^

The Patient-Centered Communication (PCC) 7-item survey^27^ was used to evaluate participants’ experience regarding CRC screening communication. The instructions indicated: “When thinking about the conversations you had about your colon health screening with your healthcare team, how often did they do each of the following?” Each statement was on a four-point Likert-type scale and ranged from Never = 1 to Always = 4. Total scores were averaged, with higher scores indicating greater PCC. Distrust in the Healthcare System was measured using a 4-item scale.^28^ For each statement, participants were asked to rate the degree they agreed using a four-point Likert-type scale ranging from “Strongly agree”=4 to “Strongly disagree”=1. The items were recoded so higher scores indicated greater distrust, and total scores were averaged.

Participants were also asked about their experience with CRC screening (never discussed, recommended but not completed, previously screened but not up to date, or up to date). Participants who reported completing CRC screening were asked what type of screening they completed (FIT, Cologuard, Colonoscopy, and I am not sure) and their likelihood to complete screening again in the future, ranging from “I definitely will check my colon health (get screened) again” to “I will not check my colon health (get screened) again.”

### Data Analysis.

We used descriptive statistics for demographic characteristics and experiences related to CRC screening, PCC, and distrust in the healthcare system. Chi-square tests and trend tests were used to evaluate the associations between sociodemographic factors and patients’ experience of CRC screening. Chi-square tests and t-tests were used to describe the association between race and ethnicity with PCC, distrust, and health literacy. The linear-by-linear trend test was evaluated using *nptrend* in Stata 18, which is a nonparametric test to detect trends in continuous variables by an ordered categorical variable. We used IBM SPSS Statistics (Version 29) to evaluate the associations between sociodemographic factors and experience with CRC screening with p-values .20 or lower were included in the regression model. We also controlled for sex in the final model and excluded preferred language as 85% of the Hispanic patients preferred Spanish. An ordered logistic regression was not appropriate as the variables age, and race and ethnicity violated the proportional odds assumption. Therefore, a binomial logistic regression was performed to explore which demographic and patient-reported factors were associated with history of CRC screening (no history vs a history of CRC screening).

## Results

Participants’ mean age was 57 (SD = 8.4; range 45–75 years old; Table 1), and the majority were Black (77%; n = 156), female (62%; n = 126), and had a high school diploma or less formal education (57%; n = 116). About half of patients had public insurance (46%; n = 94), and 20% (n = 41) completed the survey in Spanish. Regarding health literacy, 17% (n = 35) reported they were not at all or a little confident filling medical forms, and 34% (n = 70) reported at least occasional difficulty reading hospital materials.

Patients reported generally high levels of PCC about CRC screening with their providers (*M* = 3.59; SD = 0.59), and low distrust in the healthcare system (*M* = 2.03; SD = 0.45). Only 14% (n = 28) of the patients reported that CRC screening was never discussed by their doctor, 36% (n = 74) indicated that it was recommended by their doctor but not completed, 15% (n = 31) indicated that they completed CRC screening in the past but were not up to date with screening, and 35% (n = 71) were up to date with screening (Table 1).

Significant differences in CRC screening experiences were identified for age, race and ethnicity, preferred language, literacy, and PCC (Table 1). There was a significant increasing trend in age, health literacy (i.e., not needing help reading hospital materials), and PCC associated with higher chances of CRC screening. Notably, the proportion of Hispanic patients (35%; n = 17/48) who reported no discussions about CRC screening with their providers was greater than Black patients (7%; n = 11/156). Hispanic patients reported significantly lower PCC scores (*M* = 3.43, SD = 0.67) than Black patients (*M* = 3.64, SD = 0.56; p = 0.007), and no significant differences were found for self-reported medical distrust (Hispanic *M* = 2.05, SD = 0.45; Black *M* = 1.98, SD = 0.45). Hispanic patients also reported significantly lower levels of health literacy (Confident filling medical forms *M* = 3.27, SD = 1.53; Does not need help reading hospital materials *M* = 2.94, SD = 1.73) than Black patients (Confident filling medical forms *M* = 3.99, SD = 1.27; Does not need help reading hospital materials *M* = 3.97, SD = 1.34; p = 0.01). Among participants with a history of CRC screening, 69% (n = 70) patients had a history of colonoscopy, and 86% (n = 88) reported that they would definitely complete screening again (Table 2).

### Predictors of Reported history of CRC screening

We used a multiple binomial logistic regression to explore how demographic and communication factors related to the likelihood of patients having a history of CRC screening completion (Table 3). The variables included in the final model were age, sex, race, ethnicity, health literacy, and PCC. In the model, older age, Black race, and not needing help reading hospital materials were significantly associated with having a history of CRC screening. Each one-year increase in age was associated with 8% higher odds of CRC screening (OR = 1.08, 95% CI: 1.04, 1.12). Hispanic participants had lower odds of having a history of CRC completion (OR = 0.44, 95% CI: 0.20, 0.97) when compared to Black participants. Participants who reported needing less help reading hospital materials had higher odds of a CRC screening history (OR = 1.30, 96% CI: 1.01–1.67). Sex, confidence in filling out medical forms, and PCC were not significantly associated with CRC screening history in the final model.

## Discussion

This study evaluated the factors associated with experiences of CRC screening communication and completion among Black and Hispanic patients. We found that primary care patients who were younger, Hispanic, and with lower health literacy had a lower odds of reporting CRC screening.

In this study, Hispanic and Spanish-speaking patients (85% of the Hispanic patients were Spanish-speaking) had greater proportions reporting never discussing CRC screening with their providers, and lower completion rates compared to Black patients, highlighting how language continues to be a significant modifiable communication barrier to receiving recommended care.^29, 30^ Our finding is supported by a national survey from 2010–2018, where Hispanic individuals had a lower prevalence of CRC screening (52%) than Black individuals (60%). Furthermore, in the same study, foreign-born Hispanic patients with less than 15 years in the US had the lowest prevalence of CRC screening (34%);^29^ in our study, we did not collect place of birth and therefore could not compare our population to this statistic. Additionally, data from 686 community health centers across the US support that Spanish-speaking Hispanic individuals have lower odds of CRC screening than English-speaking Hispanic individuals.^30^ To address language barriers, prior studies have found that patient navigation in Spanish language improves CRC screening among Spanish-speaking patients.^31–33^ Thus, strategies such as patient navigation in Spanish-language should be implemented to increase screening rates in this underserved population.

Health literacy was significantly related to CRC screening in our study, but the broader literature is less clear.^17^ A prior systematic review found inconsistent patterns in the association between health literacy and CRC screening decision making (i.e., knowledge and attitudes); most of the studies found either no association or an association in the expected direction.^34^ In this study, we found that in the final model, health literacy was significantly associated with CRC screening, but only participants’ ratings of needing help when reading hospital materials, not their perceived self-efficacy in filling out medical forms. Prior work has also found that the health literacy questions about practical experiences are more effective in detecting inadequate health literacy.^25^ In this study, Hispanic patients reported lower levels of health literacy and PCC, suggesting the role of communication in their preferred language and acculturation might be playing in patients’ communication experiences and access to care.^35^ Thus, including practical health literacy questions as part of primary care could better identify patients who might need extra support to complete CRC screening. Additional research is needed to understand what the most important aspects of health literacy skills are for CRC screening, and how to best intervene, particularly for Hispanic patients.^36^

Previous research finds that providers’ recommendations are the major motivator for CRC screening decision-making.^37, 38^ In our study, 14% of the participants reported that their providers never recommended CRC screening, and this was associated with lower levels of PCC. In a prior study, 31% of Black participants reported their providers never suggested sigmoidoscopy or colonoscopy, and participants who reported that their provider recommended colonoscopy were 49 times more likely to complete screening than those whose provider never recommended it.^38^ Similarly, another study in primary care found that CRC screening was mentioned at 48% (48/100) of the observed visits, but no further discussion about screening occurred in half of the visits (23/48).^39^ They also found that patients initiated the CRC screening conversation in 40% (19/48) of the visits where CRC screening was mentioned without a response from the healthcare team,^39^ supporting our reported gaps in providers’ CRC screening recommendations. Another observational study supports that patients in clinical encounters with high-quality communication – as rated by independent coders – are more likely to have completed CRC screening 6 months later,^5^ highlighting the importance of high-quality PCC for screening uptake. Future intervention work could include nudges to promote provider initiation of CRC screening conversations, to ensure all patients receive CRC screening recommendations according to national guidelines.

U.S. national surveys demonstrate that PCC is related to preventive behaviors, including CRC screening.^40–42^ This is particularly relevant for Hispanic and Black patients, as a study found that Hispanic and Black patients who reported that their provider always demonstrated the domains of patient-provider communication had greater odds of CRC screening completion. ^41^ However, another national study from 2014, which used the PCC 7-item scale, found that PCC was not associated with CRC screening status.^43^ Similarly, while our study observed a significant positive trend of PCC related to CRC screening experience in discussion and completion, in the adjusted model of CRC screening history, PCC was no longer significant. While these findings highlight the importance of PCC in CRC screening communication, additional factors beyond what was covered in the PCC 7-item scale might influence whether patients complete CRC screening. Though most measures of communication tend to focus on “tasks” (i.e., did aspects of communication occur), other measures that incorporate factors such as how communication facilitates strong clinician-patient relationships or respects patients’ autonomy are strong predictors of screening behavior ^5^. Given the existing evidence of disparities in clinical communication,^10–14^ it is essential for future work to identify ways to promote equitable, high-quality communication about CRC screening among all patients, regardless of race or ethnicity.

## Limitations

This study explored cognitive, demographic, and CRC screening communication perceptions in relation to CRC completion among Hispanic and Black patients who had a recent primary care visit. Due to the high volume of potential participants, outreach was generally limited to one outreach attempt, resulting in a completion rate of 22%, which may bias the results towards patients with more positive experiences with the healthcare system or those who do not screen incoming phone calls. Nevertheless, when compared with national trends, our study exceeded the expected response rate of 6% for telephone surveys ^44^. We aimed to reach 200 patients to explore emergent patterns, rather than conduct hypothesis testing; thus, we may have been underpowered to detect significant differences by communication or other variables. Finally, patients’ CRC screening experiences were based on self-reported data and were not independently validated by electronic medical record data or direct visit observations. Further studies might help elucidate important factors for introducing and discussing CRC screening with patients, particularly among historically marginalized groups.

## Conclusion

We found that patients who are younger, Hispanic, and have lower health literacy had lower CRC screening completion, and that communication plays an important role in CRC outcomes. These results underscore the need for interventions that enhance patients’ literacy support and tailor outreach to younger, Hispanic, and low health literate populations to increase CRC screening uptake. Future studies should provide evidence-based interventions such as navigation for patients with limited health literacy and language barriers.

## Figures and Tables

**Table 1:** Patient’s characteristics by history of CRC screening

Characteristics	Total (n = 204)	No history of CRC screening discussion (n = 28) [n (%)]	Doctor suggested but never screened (n = 74) [n (%)]	History of screening but not up to date (n = 31) [n (%)]	Up to date with screening (n = 71) [n (%)]	*p-value* ^a^
Age [Mean; SD]	57.21; 8.41	56.43; 9.69	54.65; 7.13	60.77; 8.25	58.62; 8.48	.01^b^
Sex						.21
Female	128 (62.7)	18 (14.1)	42 (32.8)	17 (13.3)	51 (39.8)	
Male	76 (37.3)	10 (13.2)	32 (42.1)	14 (18.4)	20 (26.3)	
Ethnicity and Race (From medical record)						<.001
Hispanic	48 (23.53)	17 (35.42)	16 (33.33)	1 (2.08)	14 (29.17)	
Black non-Hispanic	156 (76.47)	11 (7.05)	58 (37.18)	30 (19.23)	57 (36.54)	
Preferred Language						<.001
English	163 (79.90)	15 (9.20)	60 (36.81)	30 (18.40)	58 (35.58)	
Spanish	41 (20.01)	13 (31.71)	14 (34.15)	1 (2.44)	13 (31.71)	
Education						.71^c^
< High school diploma	27 (13.24)	4 (14.81)	11 (40.74)	4 (14.81)	8 (29.63)	
High school diploma	89 (43.63)	15 (16.85)	32 (35.96)	16 (17.98)	26 (29.21)	
Some college/technical education	41 (20.10)	6 (14.63)	16 (14.63)	5 (12.20)	14 (34.15)	
College graduate	45 (22.06)	3 (6.67)	15 (33.33)	6 (13.33)	21 (46.67)	
Prefer not to answer	2 (0.98)					
Insurance						.39^d^
Private	105 (51.5)	15 (14.3)	42 (40.0)	12 (11.4)	36 (34.3)	
Public only	94 (46.1)	12 (12.8)	30 (31.9)	18 (19.6)	34 (36.2)	
Medicare only	25 (12.3)	3 (12.0)	7 (28.0)	7 (28.0)	8 (32.0)	
Medicaid only	46 (22.6)	6 (13.0)	15 (32.6)	8 (17.4)	17 (37.0)	
Medicare and Medicaid	23 (11.3)	3 (13.0)	8 (34.8)	3 (13.0)	9 (39.1)	
Self-pay	5 (2.5)	1 (20.0)	2 (40.0)	1 (20.0)	1 (20.0)	
**Health Literacy**						
Confident filling medical forms [Mean; SD]	3.82; 1.37	3.64; 1.48	3.76; 1.29	3.68; 1.47	4.03; 1.36	.16^b^
Does not need help reading hospital materials [Mean; SD]	3.73; 1.50	3.21; 1.66	3.59; 1.50	3.81; 1.33	4.03; 1.47	.01^b^
Patient-Centered Communication about CRC screening [Mean; SD]	3.59; 0.59	3.19; 0.53	3.64; 0.06	3.65; 0.57	3.67; 0.56	.007^b^
Distrust in the healthcare system [Mean; SD]	2.03; 0.45	2.02; 0.40	2.01; 0.48	2.21; 0.51	1.99; 0.40	1.00^b^

a Chi-square p-value, unless otherwise specified

b Trend test

c Minimum expected count for 1 cell was less than 5

d Only includes Private and Public only categories for data analysis.

**Table 2:** Type of CRC screening and likelihood of getting screening again by race and ethnicity among patients with a history of CRC screening (n = 102)

Variables	Black (n = 87) n (%)	Hispanic (n = 15) n (%)	Total [n (%)]
Type of CRC screening completed			
Stool test	28 (32.18)	1 (6.67)	27 (28.43)
Colonoscopy	52 (59.77)	14 (93.33)	66 (64.70)
Stool test and Colonoscopy	4 (4.60)	0 (0.00)	4 (3.92)
Don’t know	3 (3.45)	0 (0.00)	3 (2.94)
Likelihood of getting CRC screening again			
Definitely	74 85.06)	14 (14.15)	88 (86.27)
Probably	10 (11.49)	1 (6.67)	11 (10.78)
Probably not	3 (3.45)	0 (0.00)	3 (2.94)
Definitely not	0 (0.00)	0 (0.00)	0 (0.00)

**Table 3:** Multiple logistic regression model of history of CRC screening (n = 204)

Variables	OR (95% CI)
**Age**	**1.08 (1.04, 1.12)**
Woman (ref. Man)	1.55 (0.82, 2.94)
**Hispanic (ref. Black)**	**0.44 (0.20, 0.97)**
Confident filling medical forms	1.00 (0.77, 1.29)
**Does not need help reading hospital materials**	**1.30 (1.01, 1.67)**
Patient Centered Communication	1.47 (0.86, 2.51)
